# Examining the Correlation between the Inorganic Nano-Fertilizer Physical Properties and Their Impact on Crop Performance and Nutrient Uptake Efficiency

**DOI:** 10.3390/nano14151263

**Published:** 2024-07-28

**Authors:** Nothando Clementine Madlala, Nokuthula Khanyile, Absalom Masenya

**Affiliations:** 1 School of Agricultural Sciences, University of Mpumalanga, Mbombela 1200, South Africaabby.masenya@ump.ac.za (A.M.); 2 School of Chemical and Physical Sciences, University of Mpumalanga, Mbombela 1200, South Africa

**Keywords:** nano-fertilizers, surface area, nutrient use efficiency, physical properties, agglomeration

## Abstract

The physical properties of nano-fertilizers (NFs) are important in determining their performance, efficacy, and environmental interactions. Nano-fertilizers, due to their small size and high surface area-to-volume ratio, enhance plant metabolic reactions, resulting in higher crop yields. The properties of nano-fertilizers depend on the synthesis methods used. The nanoparticle’s nutrient use efficiency (NUE) varies among plant species. This review aims to analyze the relationship between the physical properties of NF and their influence on crop performance and nutrient uptake efficiency. The review focuses on the physical properties of NFs, specifically their size, shape, crystallinity, and agglomeration. This review found that smaller particle-sized nanoparticles exhibit higher nutrient use efficiency than larger particles. Nano-fertilizer-coated additives gradually release nutrients, reducing the need for frequent application and addressing limitations associated with chemical fertilizer utilization. The shapes of nano-fertilizers have varying effects on the overall performance of plants. The crystalline structure of nanoparticles promotes a slow release of nutrients. Amorphous nano-fertilizers improve the NUE and, ultimately, crop yield. Agglomeration results in nanoparticles losing their nanoscale size, accumulating on the outer surface, and becoming unavailable to plants. Understanding the physical properties of nano-fertilizers is crucial for optimizing their performance in agricultural applications.

## 1. Introduction

**Figure 1:**
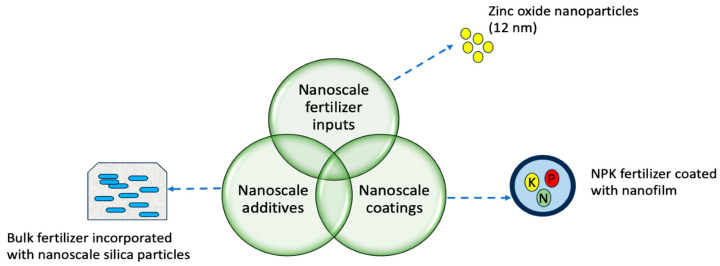
Three main types of nano-fertilizers based on their formulations.

In recent years, the utilization of nano-fertilizers (NFs) has gained significant attention in the field of agriculture. These innovative fertilizers offer a promising solution to enhance plant nutrient uptake by employing precisely formulated delivery mechanisms [1]. Nano-fertilizers possess dimensions ranging between 1 and 100 nm [2] and exhibit distinct physicochemical properties that distinguish them from traditional bulk materials [3,4]. One of the notable distinguishing characteristics of nanoparticles, in comparison to their larger counterparts composed of identical material, lies between surface effects and quantum phenomena. These two factors contribute to the unique properties exhibited by nanoparticles [3,4]. The small particle size of nanomaterials results in high surface energy, spatial confinement, and high surface area. The size and surface area of any material determine how it interacts with any biological system [5]. The adoption of this innovative approach for agricultural improvement is increasingly gaining momentum as a viable alternative to traditional fertilizers [6,7,8]. The classification of nano-fertilizers is primarily based on their formulation, which can be categorized into three main types, as depicted in Figure 1.

Nanoscale fertilizers are composed of nanoparticles that contain nutrients [9]. These fertilizers have been engineered or synthesized to contain particles or an emulsion at the nanoscale level [10]. Nanoscale additives are materials that incorporate nanoscale particles or substances into larger-scale products or inputs. These additives are not intended to serve as direct nutrients but rather to enhance the properties of the larger inputs. Nanomaterials are utilized in a limited manner within this context, substituting a small portion of larger macroscale inputs. Their main purpose is to improve the overall performance or characteristics of the final product [10]. Nanoscale coating fertilizers involve encapsulating macroscale fertilizers with a nanoscale coating or film. The film potentially contains nanoscale pores that gradually release soluble nutrients [10].

**Figure 2:**
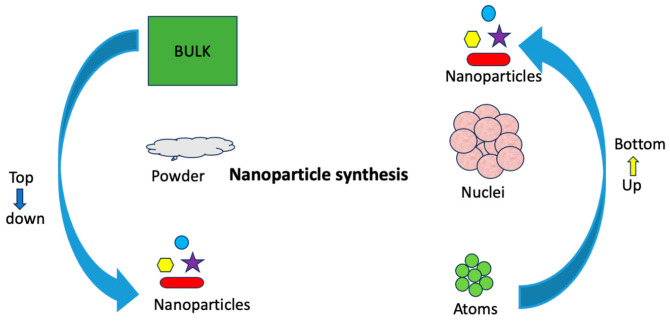
The bottom-up and top-down approach of synthesizing nanoparticles.

Nanoparticles can be synthesized using both the top-down and bottom-up approaches (Figure 2). The top-down approach involves breaking down larger particles into smaller nanoparticles through the application of mechanical forces [11,12]. In contrast, the bottom-up approach uses chemical processes to build up nanoparticles from atomic molecules [13]. The choice of synthesis method is critical as it influences the morphology, size, dispersion, and shape of nanoparticles, which subsequently affect the overall performance of the nanoparticles [14]. The bottom-up approaches, namely double emulsion-solvent evaporation and nano crystallization, produce solid nanostructures characterized by spherical shape with narrow size distribution [15]. In contrast, the top-down approach of high-pressure homogenization techniques can result in nanoparticles with irregular shapes and wide size distribution [16]. In addition, the synthesis method impacts the biocompatibility and stability of nanoparticles [17].

Nutrient uptake efficiency (NUE) is greatly influenced by fertilizer management, and its primary aim is to optimize the overall performance of crops by ensuring that the crop receives optimum nourishment [18]. The nature of nanoparticles influences the uptake of nutrients [19], and every plant species is unique and possesses its own optimum nutrient range and a minimum requirement level [20]. Plants exhibit symptoms of nutrient deficiency when they receive nutrients below their minimum nutrient requirement. Concurrently, excessive nutrient uptake potentially results in an imbalance that, in turn, results in poor plant growth and toxicity [21]. Therefore, it is imperative to closely observe the assimilation and translocation of nutrients to prevent the occurrence of both nutrient toxicity and deficiency in crops.

There are multiple ways in which nanoparticles can enter the plant system, including through root hairs, cracks on the leaf surface, and stomata [2]. There are numerous methods for delivering nanoparticles, including root application, feeding/injecting directly into plant tissue, and foliar application [22]. Nanoparticles can traverse the plant system via bulk flow, phloem loading, and diffusion after entering the plant [23]. Understanding the mechanism by which plants absorb and transport nano-fertilizers is imperative for the development of the most efficient formulations [24]. Examining the mechanism of action and bioaccumulation of these nano-fertilizers may provide valuable insights regarding their biological safety and recommendations for their safe use [25]. This review seeks to evaluate the relationship between the physical properties of nano-fertilizers and their performance by exploring how these unique properties influence factors such as plant response, nutrient uptake, and plant growth parameters.

## 2. Search Strategy

All articles and studies were identified based on ScienceDirect, ResearchGate, and Google Database searches dating from 2008 to 2024. The keywords and phrases in relation to this review article include: “size of nano-fertilizers, shape of nano-fertilizers, high surface area of nano-fertilizers, slow-release of nano-fertilizers, nutrient uptake of nano-fertilizers, agglomeration of nanoparticles, crystalline structure of nano-fertilizers, amorphous nano-fertilizers, and charges of nano-fertilizers”. In total, 148 relevant articles were selected.

### 2.1. Synthesis Methods of Nano-Fertilizers

The synthesis of nanoparticles (NPs) is paramount in determining their properties, such as shape, stability, size, and surface characteristics [26]. Several synthesis techniques are used to tailor these properties for specific applications, and understanding these methods is essential for optimizing nanoparticle performance in agriculture [27]. Particle size can vary depending on the method used for synthesizing nanoparticles. For example, the study by Kathad and Gajera [28] compared the synthesis of copper nanoparticles using biological and green chemistry methods. Their findings indicated that the green chemistry method, which used ascorbic acid as a reducing agent and Cetyltrimethylammonium bromide for size control, produced nanoparticles with an average size of 35 nm. On the other hand, the biological approach utilized plant extracts from *Artabotrys odaratissimus*, resulting in larger nanoparticles with an average of 135 nm. In addition, Lu et al. [29] synthesized highly stable and well-dispersed copper nanoparticles with a particle size of 34 nm through a two-step synthesis method that uses non-toxic ascorbic acid as a reducing agent and polyvinylpyrrolidone (PVP) as a coating agent.

Furthermore, Taha et al. [30] used a novel hydrothermal pyrolysis method, which resulted in nanoparticles with sizes increasing from 58 nm to 108 nm as the reaction time increased, indicating the capability of the method to control particle size through reaction time adjustments. Moreover, Fokina et al. [31] discovered that the use of specific solvents such as docosane, 1-octadecene, and trioctylamine enabled the production of monodisperse iron oxide nanoparticles ranging between 6 and 24 nm. The researchers achieved reproducible size control by regulating the temperature within the range of 300 to 370 °C and using a thermal pretreatment of the iron oxide precursor. By adjusting parameters such as precursor concentration, temperature, and heating rate, the researchers consistently produced iron oxide nanoparticles with small particle sizes. Thus, the choice of synthesis method significantly influenced the size of the nanoparticles produced.

Researchers have reported that the biological methods of synthesizing nano-fertilizers effectively prevent agglomeration via various mechanisms that leverage the unique characteristics of biological processes and materials. For example, de França Bettencourt et al. [32] discovered that the use of bacterial supernatants containing auxin complexes, such as indole-3-acetic acid (IAA), act as capping and reductive agents during the synthesis of manganese and iron nanoparticles. This biological capping prevents the nanoparticles from clustering together, thereby enabling them to be well-dispersed and effective as micronutrient nano-fertilizers. The biological synthesis of nanoparticles, specifically the use of plant extracts, prevents agglomeration due to the presence of several phytochemical compounds, including amino acids, alkaloids, phenols, flavonoids, and proteins. These compounds produce nanoparticles with small particle sizes and enhance their stability by capping the nanoparticles, thus preventing agglomeration [33]. For example, Dallatu et al. [34] used Azadirachta indica seed husk extract in green synthesis of zinc oxide nanoparticles and found that more of the extract led to the formation of smaller, more uniform nanoparticles that did not stick together. This was confirmed by TEM analysis.

The synthesis method of nanoparticles influences the incorporation of nutrients and their release, affecting their efficacy as nano-fertilizers. Various synthesis methods, such as chemical, biological, and physical techniques, each have a unique impact on the properties of nanoparticles, including their release kinetics and nutrient loading [35]. A study conducted by Tombuloglu et al. [36] synthesizes composite micro-nutrient nanoparticles (nickel, zinc, iron, and copper) through the sol-gel auto-combustion method. The findings indicated that the nutrient elements were effectively incorporated into plant tissues, with different concentrations affecting nutrient uptake in the leaves and roots of barley. This indicates that the synthesis technique significantly influences how nutrients are incorporated into nanoparticles and subsequently released into plants. The use of mesoporous silica nanoparticles and layered double hydroxide in the synthesis of nano-enabled fertilizers has revealed that the release kinetics of nutrients heavily rely on the shape, composition, and size of nanoparticles, as well as the environmental conditions. Thus, these factors influence the nutrient use efficiency [37]. It is imperative to select the appropriate synthesis method to optimize the physical properties and performance of nano-fertilizers.

### 2.2. The Particle Size of Nanoparticles

**Figure 3:**
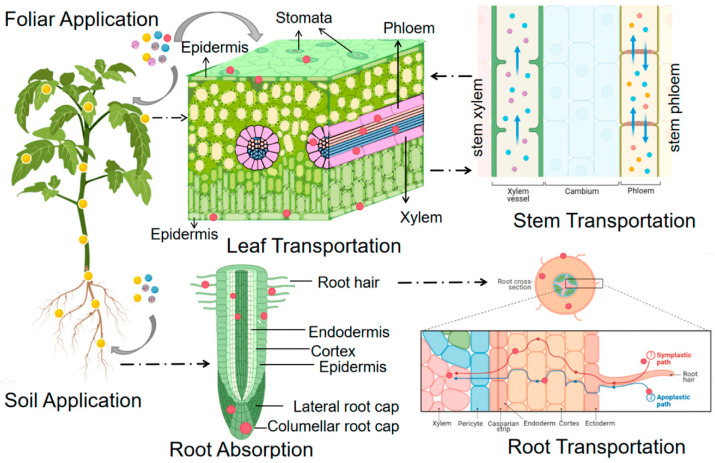
Schematic diagram showing the routes of nanoparticles through roots and leave “Image adapted from Wang et al. [18], *Wang P, Yin H. Nanoparticles in plants: uptake, transport and physiological activity in leaf and root. Materials,* [MDPI], [2023]. Permission to produce this image was not requested.

Nanoparticle size is a critical physical property that significantly impacts nutrient use efficiency and crop performance [37]. Ensuring the entry of nanoparticles into plant tissues is critical because it allows them to be available for the plants’ metabolic processes [38]. Several pathways and mechanisms can influence the entry routes of nanoparticles into plant systems, which determine the size of the particles [25]. Plants can absorb nanoparticles through their roots, which then traverse the epidermal layers and move into the xylem for transport to the plant’s aerial parts. Another common route is foliar entry, where nanoparticles can penetrate through stomata or cuticular pores [39,40] (refer to Figure 3). The cellular entry of nanoparticles is impacted by their size, which determines their translocation pathways, such as inner attract, free translocation, outer wrapping, and embedment [41].

**Figure 4:**
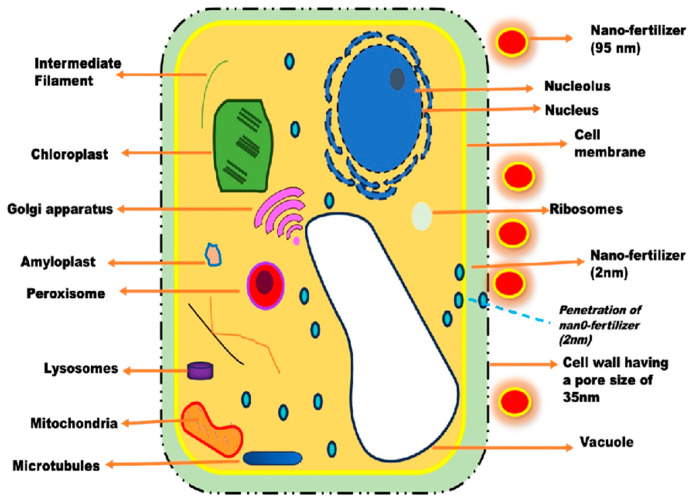
Depiction of a schematic representation of a plant cell. The representation illustrates a plant cell wall with a pore size of 35 nm, and this size restricts any material above 35 nm to only material with a pore size less than 35 nm. The nano-fertilizer with a particle size of 2 nm was able to penetrate the plant cell wall, while the nano-fertilizer with a particle size of 95 nm was unable to penetrate due to the cell wall pores being 35 nm.

Nanoparticles with smaller sizes have been observed to successfully pass through the cell wall pores and enter the cell membrane [42,43]. In contrast, nanoparticles that exceed the size of the cell wall pores have been found to accumulate outside the cell wall because they are unable to penetrate and enter the cell [44,45] (Figure 4). Researchers have reported that small-sized nanoparticles, owing to their larger surface area, are more reactive and can easily enter plant cell membranes and cell walls, facilitating their movement through symplastic and apoplastic pathways [46].

Qadir and Fathulla [47] examined the impact of nickel nanoparticles of different sizes (20, 40, and 70 nm) on *Phaseolus vulgaris* plants. The findings indicated that as the nanoparticle size increased, the accumulation of nickel in the roots and shoots also increased, demonstrating that they impeded the outer surface of the plant. In addition, the larger particles (70 nm) led to a reduction in shoot and root biomass. However, the small particles (40 nm) improved the chlorophyll and pigment content of the plant. Similarly, Hu et al. [48] conducted hydroponic experiments with the aim of examining the absorption of selenium nanoparticles (SeNPs) in wheat plants. The researchers synthesized SeNPs with varying dimensions, specifically 40 nm, 140 nm, and 240 nm. Subsequently, they conducted an analysis to examine the absorption properties of these nanoparticles. The research results indicated that the uptake of SeNPs by wheat roots was influenced by the size of the particles. The absorption of 40 nm SeNPs was found to be 1.8–2.2 times higher compared to that of 140 nm and 240 nm SeNPs. The study revealed that once absorbed by wheat plants, selenium nanoparticles with a size of 40 nm were quickly oxidized to selenite, which is a more reactive form. The selenite was then transformed into organic selenium compounds such as selenomethionine, selenocystine, and se-methyl-selenocysteine. Samynathan et al. [49] reported that selenomethionine, selenocystine, and se-methyl-selenocystein enhance plant metabolism, increase resistance to biotic and abiotic stresses, and promote plant development.

Yusefi-Tanha et al. [50] aimed to investigate the effect of copper oxide nanoparticles (CuONPs) on soybean plants and the implications for human health. Over the course of a comprehensive 120-day study, the effects of CuONP particles of varying sizes (25 nm, 50 nm, and 250 nm) on root system architecture, soil–root interface, and Cu transport and accumulation were examined. The results highlighted that higher copper uptake was observed for CuONPs with a particle size of 25 nm compared to the nanoparticles containing 50 nm and 250 nm. The study demonstrated the smallest copper oxide nanoparticles (25 nm) had a notable impact on root biomass, area, length, and volume. These nanoparticles (25 nm) were found to have a greater inhibitory effect on root growth compared to the larger particles. However, these nanoparticles (25 nm) increased the total copper content in seeds, which could potentially enhance their nutritional value.

In a related study, Zhang et al. [51] set out to explore how ceria nanoparticles (ceria NPs) are absorbed and distributed within cucumber plants. The researchers prepared two different sizes of ceria nanoparticles, measuring 7 nm and 25 nm, respectively. Their results revealed that cucumber roots exhibited a higher uptake of 7 nm ceria nanoparticles compared to the larger 25 nm particles. Inside the roots, the 7 nm ceria nanoparticles moved through the vascular system, indicating a great barrier. The distribution studies demonstrated that once in the vascular cylinder, the nanoparticles effortlessly traveled to the end of the vascular bundle, facilitated by the plant’s water transport mechanisms.

Kumar et al. [6] conducted research that aligns with the earlier findings of Yusefi-Tanha et al. [50], where they observed that nano-urea, also known as nano nitrogen, possesses the ability to efficiently penetrate plant cell walls and reach the plasma membrane. This capability is attributed to the small particle size of nano-urea, which typically falls within the range of 18 to 30 nm. Another study by Gordillo-Delgado et al. [52] explored how silver-incorporated titanium dioxide nanoparticles (Ag-TiO_2_ NPs) impacted the physiology and growth of spinach seedlings. The findings of the study revealed that plant development was enhanced by smaller particle size of 7–26 nm, as evidenced by an increase in photosynthetic activity. In contrast, the larger particle size of 43 nm did not improve plant growth. This is because the nanoparticles clustered together, blocking the roots pores and causing a decrease in germination rate and water absorption.

Based on the data presented in Table 1, it can be inferred that there is a positive correlation between smaller particle sizes and increased nutrient uptake compared to larger particles. Plants easily absorb nanoparticles that are small in size, while they poorly absorb those that are large in size. The nano-fertilizers that contain 20 nm particles exhibit a higher uptake of nutrients from gold nanoparticles in watermelon compared to those with a size of 60 nm. Similarly, the 3.5 nm gold nanoparticles exhibit a greater uptake in *Nicotiana xanthi* compared to the 18 nm nanoparticles. It has been observed that all the small-sized nanoparticles mentioned in the table are easily absorbed compared to their bulky or large counterparts.

It is important to recognize that the morphology of plants varies, and this variation has a significant impact on nutrient absorption. The uptake of nanoparticles is influenced by the pores in the plant cell wall, and different plants have different types of cell wall pores. These pores act as barriers for the plant cell, preventing materials larger than the size of the plant cell wall pores from entering the cell. They accumulate on the outer surface of the plant cell and are not easily accessible for the plant’s utilization. This statement is in agreement with the report by Carpita et al. [55]. In their study, researchers found that the diameter of pores in the cell wall of *Raphanus sativus* roots ranged from 3.5 nm to 3.8 nm. The limiting diameter for Gossypium hirsutum fibers was found to be between 3.8 nm and 4.0 nm. According to their findings, particles larger than the determined diameters were unable to penetrate the cell. It has been observed that watermelon cells can allow the penetration of gold nanoparticles, which are smaller than 20 nm in size, through their cell walls.

However, it has also been observed that in *Nicotiana xanthi*, gold nanoparticles larger than 18 nm were unable to penetrate the cells. The cells of *Nicotiana xanthi* only allow nanoparticles that are smaller than 3.5 nm in size. Therefore, it is important to consider the size of the plant cell membrane when applying nanoparticles. It is crucial to ensure that the nanoparticles are smaller than the pores to effectively penetrate the membrane. This will help prevent the accumulation of nutrients on the cell membrane’s outer surface. Maximizing the size of nanoparticles for agricultural applications not only optimizes plant nutrient uptake and development but also emphasizes the complex relationship between plant physiology. This paves the way for more efficient and sustainable farming practices.

For nanoparticles to be effective, it is crucial that they can be easily absorbed and made available for plant utilization. Nano-fertilizers can penetrate the plant system either through the roots or leaves. The small size of nanoparticles has been shown to have the ability to enter plants and enhance their biochemical and physiological processes, while larger particles are unable to penetrate the plant system. Understanding the entry of nanoparticles into plant cells is crucial as it correlates directly to plant uptake efficiency of nutrients. The size of the particles plays a significant role in determining whether they can penetrate the plant cell wall and membrane. If the particle size exceeds that of the plant cell wall, the nano-fertilizer will not be able to enter the plant. It is important to note that different plant species have varying cell wall pores due to differences in their morphology. As a result, the size of the particles needed for penetration will vary depending on the plant species. Understanding this property of nano-fertilizers will allow for the development of nano-fertilizers with particle sizes smaller than the cell wall and membrane of the targeted plants, ensuring efficient absorption of the nanoparticles by the plants.

### 2.3. The Surface Area-to-Volume Ratio of a Nanoparticle

**Figure 5:**
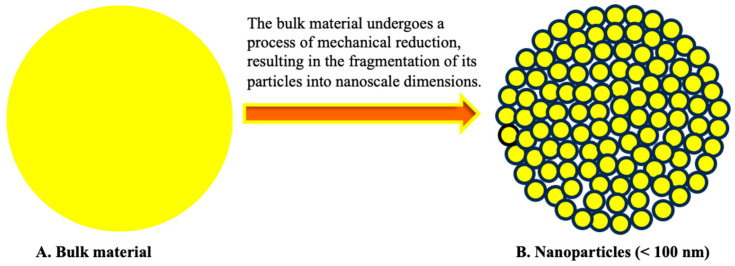
The relationship between particle size and surface area.

Nanoparticles possess a remarkable characteristic known as a high surface area-to-volume ratio due to their small size [56,57]. The surface area refers to the complete outer covering of a material [58], while the volume represents the amount of space occupied by the material [59]. The high surface area is a significant physical property of nanoparticles [60], and it plays a crucial role in various fields, including medicine and pharmaceuticals, agriculture, the food industry, electronics, chemical catalysis, and many others [61]. It has been observed that there is a relationship between the surface area-to-volume ratio of nanoparticles that is dependent on their size. The smaller the size of the particles, the greater the surface area. On the other hand, as the particle size increases, the surface area-to-volume ratio decreases [62]. Figure 5 depicts two materials that demonstrate a correlation between particle size and the surface area-to-volume ratio. One material has larger particles (bulk material) and a lower surface area, while the other material consists of smaller particles (nanoparticles) with a higher surface area. The volume of the two materials remains constant.

Compared to their bulk counterparts, the high surface area-to-volume ratio of nanoparticles facilitates increased exposure and accessibility of active sites. This promotes interactions with other substances [63]. Due to their high surface area-to-volume ratio, nano-fertilizers offer a greater area for photosynthesis. This leads to increased absorption of sunlight and, ultimately, higher crop yields [64]. Nanoparticles encapsulating nutrient particles have the ability to retain nutrients due to their distinct surface properties. These properties enable targeted and gradual release of nutrients, unlike the conventional material surfaces used in the production of chemical fertilizers [65].

Nano-porous zeolites have been recognized as an outstanding source of slow-release nutrient fertilizers. These zeolites exhibit a distinct structure characterized by a network of interconnected pores at the microscopic level. This pore structure allows them to effectively retain nutrients and release them slowly to plants in a controlled manner. The use of nanoporous zeolites as slow-release fertilizers has numerous advantages. Firstly, it helps to reduce the loss of nutrients, which are typically prone to volatilization or leaching when conventional fertilizers are applied. Zeolites function as reservoirs by entrapping nutrients within their porous structure, ensuring their sustained availability to plants and preventing their premature loss. Furthermore, the extensive surface area and high reactivity of nanoporous zeolite make them suitable for replacing nutrients that are substituted by other ions through a cation exchange process [66]. Researchers have reported that nano-fertilizers can gradually release nutrients over a period of 40–50 days. In contrast, synthetic fertilizers achieve full nutrient release within a much shorter timeframe of 4–10 days [67].

The controlled and gradual release of nutrients through the use of nano-fertilizers has been found to improve the efficiency of nutrient utilization [68]. The manner in which nutrients are released is greatly influenced by the design of the fertilizer [69]. As a result, researchers have developed fertilizers coated with nanomaterials to ensure a gradual release of nutrients that match the specific needs of crops [70]. The data presented in Table 2 illustrate that utilizing nano-fertilizer-coated additives improves nitrogen use efficiency (NUE) by releasing nutrients gradually over an extended duration, as opposed to conventional fertilizers. The study conducted by Ghorbanpour et al. [71] reported that urea coated with nanoparticles exhibited a prolonged release of nitrogen over 50 days. In contrast, uncoated urea requires a shorter duration of 10–12 days to release nutrients.

In their study, Hidayat et al. [72] assessed the effectiveness of urea/APTMS-modified zeolite as a slow-release nitrogen fertilizer. The zeolite modified with APTMS exhibited a prolonged release of nitrogen, with a release time of 120 min (equivalent to approximately 2 h), in contrast to the rapid release of nitrogen observed with regular urea, which occurred within 10 min. The gradual release of nitrogen can be attributed to the surface modification of zeolite using APTMS. These findings are consistent with the results reported by Kottegoda et al. [73], who investigated the efficacy of urea-modified hydroxyapatite nanoparticles encapsulated under pressure into cavities of the soft wood of Gliricidia sepium. The nitrogen release of the nano-fertilizer composition was investigated by conducting a study using soil samples collected from three different elevations in Sri Lanka, with pH levels of 4.2, 5.2, and 7. Comparing the nitrogen release of the nano-fertilizer composition with that of a commercially available fertilizer, the authors observed that the nano-fertilizer exhibited an initial rapid release followed by a gradual and sustained release even on day 60. The commercial fertilizer, on the other hand, demonstrated a significant early release followed by a subsequent release of lower and uneven quantities until approximately day 30.

The rapid release of nutrients associated with conventional fertilizers has been identified as a cause of several environmental problems, including air, water, and soil pollution. This is a significant and ongoing global issue as we work towards achieving a healthy and sustainable environment [77]. There are different ways in which nano-fertilizers are engineered to release nutrients (refer to Table 3 below). The utilization of a slow-release mechanism for nutrients effectively decreases the need for frequent fertilizer application, thereby enabling farmers to mitigate the expenses associated with such regular applications [78]. Nano-fertilizers can be designed to control their nutrient release in various ways [79].

### 2.4. Shape of Nanoparticles

The shape of a material refers to its external form, outline, or contours, regardless of its actual size. However, the distinction between shape and size is unclear. Additionally, as the size of the particles decreases, the shape undergoes a transformation. This transformation primarily occurs during the process of milling and crushing [27]. Researchers have demonstrated that temperature, pH, and reaction time can influence the shape of liquid nanoparticles during the formation stage. For instance, the increase in reaction rate caused the morphology of liquid silver nanoparticles to vary with pH, indicating a relationship between nanoparticle size, reaction pH, and acid type [81]. Similarly, the pH of the precursor solution significantly influenced the shape of nanorods in the synthesis of ZnO nanostructures, while the reaction time and temperature affected the size of the nanoparticles [82].

**Figure 6:**
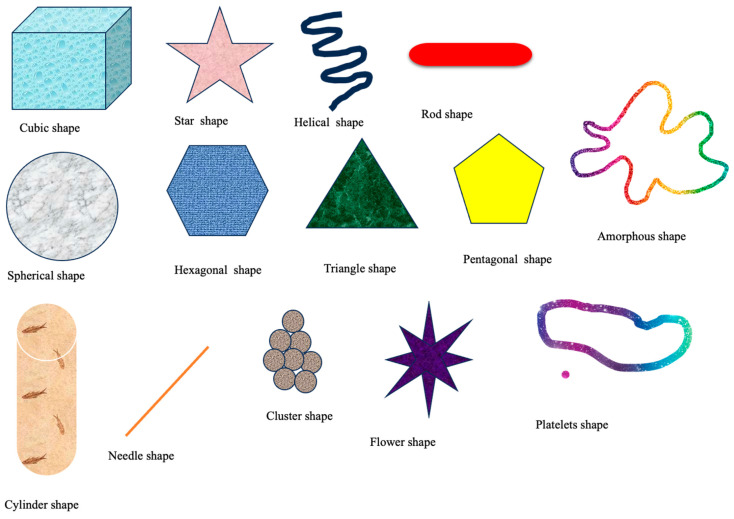
Shape of nanoparticles.

The shape of nanoparticles plays a crucial role in the synthesis of materials with desired functions [83]. The shape of nanoparticles depends on various factors, including their interaction with stabilizers and inductors, as well as the methods used to synthesize these materials [84]. Nanoparticles can take on various shapes (refer to Figure 6 below). Nanoparticles (NPs) display a wide range of interfacial properties because of their various shapes. This leads to variations in the surface area of the nanoparticles and the contact angles observed when they interact with the plant surface. These factors ultimately influence the regulation of nanoparticle absorption [85]. Researchers have found that carbon-based nanomaterials, including carbon nanotubes (CNTs), fullerenes, and graphene, possess a high surface area-to-volume ratio due to their nanoscale structure. This allows them to attract and release molecules effectively [86].

The shape of nanoparticles is characterized by using various powerful tools such as Transmission Electron Microscope (TEM), High-Resolution Transmission Electron Microscope (HRTEM), and Scanning Electron Microscope (SEM) [87]. The variations related to shape have been found to influence the absorption of nanoparticles directly [88]. Researchers have demonstrated that there is a relationship between nanoparticle shape and plant performance of various crop species (refer to Table 4 below). A study conducted by Zhang et al. [89] compared the absorption and internalization of rod-shaped gold nanoparticles and spherical nanoparticles. The results of their study showed that, even though the nanoparticles had similar sizes, the rod-shaped nanoparticles were more likely to be absorbed and taken up by Arabidopsis leaves.

It has been demonstrated that the utilization of spherical silver nanoparticles (AgNPs) at a low concentration of 60 mg L^−1^ can effectively improve multiple plant growth parameters in *Phaseolus vulgaris* [91]. The application of higher concentrations of AgNPs resulted in a decrease in the number of leaves, plant height, and root length, as observed in the study by Abd El-Aziz and Al-Othman [90]. Thus, silver nanoparticles at lower concentrations can potentially augment germination and various plant growth parameters.

In contrast, the germination percentage of spherical-shaped ZnONPs is higher at higher concentrations compared to lower concentrations. For example, when ZnONPs were applied to Blackgram at a concentration of 100 mg L^−1^, a germination rate of 67% was observed. On the other hand, 600 mg L^−1^ of the same ZnONPs resulted in the highest germination rate, reaching 74% [73]. The researchers observed the same phenomenon in the plant growth parameters, exhibiting a notable enhancement in shoot and root length when ZnONPs were administered at a concentration of 600 mg L^−1^ compared to lower concentrations of spherically shaped ZnONPs.

### 2.5. Agglomeration

Agglomeration of nanoparticles is the phenomenon in which individual nanoparticles come together to create larger clusters, also known as agglomerates [98]. Agglomerated nanoparticles can impede nutrient absorption by changing their chemical and physical properties, thus influencing their interaction with soil and plant systems. The agglomeration of nanoparticles decreases their reactivity and surface area. Thus, their ability to act as effective nutrient carriers is reduced. This clustering can prevent the movement of nanoparticles through major barriers such as roots, which in turn limits their accessibility to plants [99]. The agglomeration of nanoparticles inside plant cells can result in an uneven distribution, causing nanoparticles to remain clustered in specific areas instead of being dispersed uniformly [100]. The slow release of nutrients from agglomerated nanoparticles can be less effective compared to well-dispersed nanoparticles [101].

When nanoparticles aggregate, their distinct physicochemical properties are compromised, decreasing their ability to act as ‘magic bullets’ that target specific cellular organelles in plants [102].

Du et al. [103] discovered that TIO_2_ NPs, owing to their agglomeration status, adhered to the cell walls of the wheat plant, and they could not penetrate the roots, whereas the ZnO NPs were easily absorbed by the wheat cell and tissues. These findings underscore the crucial role of agglomeration in influencing the infiltration and behavior of nanoparticles within plant cells. The distribution of agglomerated nanoparticles can be influenced by the synthesis method selected. Bruinink et al. [99] observed that citrate-stabilized nanoparticles exhibited an even distribution on the barley leaf surface; they avoided entering the stomates, whereas plant extract-stabilized nanoparticles formed a thin layer and accumulated on all areas of the leaf, including the stomates.

To address the issue of agglomeration, researchers have proposed various strategies. One such strategy involves the manipulation of the zeta potential of nanomaterials to augment the repulsive forces acting between particles. By increasing the zeta potential, the electrostatic repulsion between particles is enhanced, thereby discouraging their aggregation. Another approach is to optimize the hydrophilicity or hydrophobicity of the nanomaterial. This can be achieved by modifying the surface properties of the particles, allowing for better dispersion and reduced tendency for agglomeration. Additionally, adjusting the pH and ionic strength of the suspension medium has been identified as a potential strategy. By carefully controlling these parameters, researchers aim to create an environment that discourages particle aggregation and promotes stability [61]. Maintaining the dispersity of nanomaterials is essential to preserve their surface effects, as strong, attractive interactions between particles can lead to agglomeration and aggregation, negatively impacting their surface area and nanoscale properties [104].

### 2.6. Crystalline Structure

A crystal structure consists of a unit cell—a set of atoms arranged in a specific pattern. This arrangement is periodically repeated in three dimensions on a lattice [105]. The crystalline structure consists of single or multi-crystal solids, but they can also be non-crystalline, which is known as an amorphous structure [106]. Starch-based nano-fertilizers consisting of nanocrystals can be readily dissolved in water [107]. Fast-dissolving fertilizers have been associated with high nutrient uptake by plants [108]. Therefore, starch-based nano-fertilizers with nanocrystal structures can have high nutrient uptake. The crystalline structure of nanoparticles influences their translocation within the plant [109]. Carmona et al. [110] found that the structure and shape of nanoparticles greatly influence their dissolution rate. Researchers found that crystalline nanoplatelets released nitrate more slowly, while spherical amorphous nanoparticles, due to their surface chemistry, exhibited fast nutrient release. Ramírez-Rodríguez et al. [111] initially synthesized nano-PK and nano-NPK, both exhibiting an amorphous calcium phosphate structure, which resulted in the rapid release of nutrients. They then doped these nanoparticles with urea to create nanoU-NPK. Researchers found that nanoU-NPK had a crystalline structure and gradually released nutrients. The study observed increased growth in durum wheat when treated with nanoU-NPK. Researchers have reported that the slow release of nutrients enables a better synchronization between nutrient availability and plant demand, leading to increased nutrient uptake and utilization efficiency [112].

Elsabagh et al. [113] demonstrated that the use of nano-sized water treatment residuals (nWTR) containing amorphous aluminum, iron, and silicon enhanced the soil properties and nutrient absorption compared to traditional fertilizers. The authors reported that the high concentration of amorphous aluminum and iron can significantly influence the absorption of potassium and phosphorus. Additionally, the presence of amorphous iron and aluminum in the soil significantly altered the ionic charge, ion adsorption, particularly for phosphorus, and the formation of aggregates and swellings. The improvement of the soil properties resulted in improved water and nutrient retention in the soil and increased the growth parameters of the maize crop compared to the traditional fertilizers. Carmona et al. [114] reported that amorphous calcium phosphate (ACP) demonstrates high solubility compared to nanocrystalline apatite (nAp) and exhibits higher surface reactivity, allowing ACP to have larger nutrient payloads compared to nAp. According to Sakhno et al. [115], amorphous calcium phosphate (ACP) has been found to be a viable substitute for conventional fertilizers. This is because ACP can be enriched with important micronutrients, has adjustable solubility for phosphorous release, and possesses a large specific surface area. In research conducted by Sakhno et al. [115], it was discovered that the use of citrate-stabilized amorphous calcium phosphate nanoparticles (ACPc) with added micronutrients (zinc, boron, magnesium, and copper) resulted in a 22% increase in lettuce crop yield compared to the use of monocalcium phosphate (MCP). The doped ACPc showed superior phosphorous use efficiency compared to MCP.

When it comes to selecting the ideal nano-fertilizer, it depends on the specific agricultural needs and desired outcomes. Researchers have reported that nano-fertilizers release nutrients gradually, ensuring a prolonged and consistent supply of nutrients. The gradual release of nutrients minimizes environmental concerns associated with a rapid release while maintaining optimal crop yield [115]. Amorphous nano-fertilizers have a rapid release of nutrients and high solubility, ensuring that nutrients are immediately available to plants. Additionally, the high surface area and higher nutrient loading capacity of these nano-fertilizers make them flexible enough for use as nutrient carriers. The high surface area of amorphous nano-fertilizers improves their reactivity and ultimately increases crop productivity [116]. However, the rapid release of amorphous nano-fertilizers is associated with environmental concerns [117]. Fast-release fertilizers can have a negative effect on aquatic ecosystems. This is because they dissolve quickly, which can result in excessive amounts of nutrients being applied to plants. As a result, these nutrients can run off into water bodies, resulting in eutrophication [118].

### 2.7. Charge Properties of Nano-Fertilizers

Nano-fertilizers commonly exhibit a surface charge that is either positive, negative, or neutral [119]. The functional groups and chemical composition present on the surface of these nanoparticles influence their charge [120]. The surface charge of nano-fertilizers plays a significant role in determining their mobility, how they interact with soil particles, and their overall ability to effectively deliver nutrients to plants. It is paramount to understand and effectively control this property to optimize the use of nano-fertilizers in agricultural applications [121]. Surface charge properties play an important role in either promoting or preventing aggregation. Nanoparticles with charged surfaces can either attract or repel each other, depending on the specific type and magnitude of the charges they exhibit [122]. Oppositely charged nanoparticles attract each other because of electrostatic forces. This phenomenon can result in the formation of aggregates [123,124]. However, nanoparticles with similar charges repel each other. This repulsion helps to enhance their dispersion in a solution [125]. Dispersed nanoparticles are desirable due to their availability to be absorbed by plants and distributed uniformly within them.

Soil particles and organic matter carry charges [126]. The interaction between soil components and the charge of nano-fertilizers is critical for plants’ nutrient release and uptake [127]. For instance, a study conducted by Huang et al. [128] explored the potential of using negatively charged nano-hydroxyapatite (nHAP) as a phosphorus fertilizer in soil contaminated with cadmium. The findings indicated that the presence of nHAP had a notable impact on the availability of phosphorus in the soil, thereby enhancing its uptake. The study showed that the negative charge of nHAP attracts positively charged phosphorus ions, making them more accessible for plant nutrition. This innovative approach presents a potential solution for improving soil fertility and promoting growth in contaminated soils.

Another study conducted by Elhaj Baddar [119] explored the impact of the surface charge of zinc oxide (ZnO) nanoparticles on zinc availability and wheat growth. The study discovered that the surface charges of ZnO nanoparticles, specifically the negatively charged DEX (SO4) and core shell ZnO nanoparticles, had a significant impact on the distribution of zinc in the soil and plant tissues. These nanoparticles with a negative charge demonstrated greater effectiveness and solubility in increasing the concentration of Zn in soil extracts at alkaline pH levels compared to positively and neutrally charged ZnO nanoparticles. The improved availability of Zn resulted in increased grain Zn concentrations and enhanced overall wheat yield, demonstrating the benefits of engineering nanoparticle surface charges to maximize their performance as nano-fertilizers.

Similarly, Devnita et al. [129] explored the influence of negatively charged rock phosphate nanoparticles on the behavior of variable charge soils, specifically andisol. The findings revealed that the use of 7.5% rock phosphate nanoparticles led to a reduction in phosphorous retention, reaching 87.22% after a span of four months. Moreover, it led to a gradual increase in the availability of phosphorous levels (245.37 mg/kg) and potential phosphorous content (1354.78 mg/100 g) as time progressed. When biofertilizers were used in combination, the retention of P showed a remarkable improvement of 91.66% after four months, despite an initial decrease in the levels of available and potential P. However, these levels later increased significantly. Additionally, after one month, the study revealed that the utilization of rock phosphate nanoparticles and biofertilizers resulted in an increase in P retention from 75 to 77%. The research findings highlight the positive impact of using negatively charged nanoparticles, such as rock phosphate in nanoparticle form, in combination with biofertilizers. This combination has been shown to significantly improve soil phosphorus availability and enhance soil fertility, particularly in variable-charge soils.

However, the study by VandeVoort and Arai [130] on copper nanoparticles presents a contrasting scenario; while negatively charged nanoparticles enhanced nutrient availability, copper nanoparticles, which are positively charged, had a more complex and generally negative effect on soil nitrification. The study found that low concentrations of copper nanoparticles mg L^−1^ could enhance nitrification rates due to their role as micronutrients. However, at higher concentrations 10–100 mg L^−1^, CuNPs led to significant suppression of the nitrification process. This suppression was attributed to the toxicity of both the CuNPs and the ionic copper (Cu^2^⁺) released from them. This study highlights that, unlike ZnO nanoparticles, the use of positively charged CuNPs needs to be carefully managed because their impact on soil microorganisms and biogeochemical processes can be detrimental at higher concentrations. It underscores the importance of considering the specific properties of nano-fertilizers, such as their charge and concentration, when evaluating their effectiveness and safety in agricultural systems.

### 2.8. Nutritional Value of Nano-Fertilizers

Researchers have extensively reported on the benefits of nano-fertilizers in enhancing agricultural productivity and addressing the limitations of conventional fertilizers [130]. However, it is imperative to contemplate the nutritional value of nano-fertilizers. Crops rich in nutrients can effectively meet the recommended dietary requirements, thus lowering the risk of nutrition-related disorders [131]. Specifically, the utilization of nano-fertilizers has been observed to induce changes in the availability of nutrients through the regulation of their quantity and quality, ultimately leading to enhancements in the nutritional composition of plants [132,133,134]. The nutritional value of nano-fertilizers lies in their ability to enhance nutrient availability to crop plants through increased surface area. Consequently, the increase in the rate of reaction or synthesis process in the plant system contributes to improving quality parameters, including protein, oil, and sugar.

The utilization of nanoformulations containing zinc and iron has been observed to result in an augmentation of various essential components within crop grains. Specifically, applying these nanoformulations has been shown to enhance the overall levels of carbohydrates, starch, indole-3-acetic acid (IAA), chlorophyll, and protein content [135]. Researchers have reported that the availability of nutrients during the growing cycle of the plant significantly influences its nutritional content [114]. For instance, Al-Juthery et al. [72] discovered nanoamino acids, and nanopotassium increased the nutritional value of wheat by increasing the concentration of essential micronutrients (Zn, Mn, Fe, and Cu) in the grain. Another study by Rahman et al. [136] revealed that applying nano-fertilizer resulted in high nutrient use efficiency and significantly improved the nutritional value of tomatoes. Thus, it is imperative to ensure that plants have access to nutrients during their growing cycle to produce food with a high nutritional value and high yield that will meet the nutritional requirements of the population.

The study conducted by ul Ain et al. [137] assessed the impact of magnesium nano-fertilizers on the nutritional composition and crop performance of green beans. The study applied magnesium nano-fertilizers at different concentrations and compared their effects to traditional MgSO_4_ fertilizers. The findings demonstrated that magnesium nano-fertilizers significantly improved magnesium content in the green beans, accomplishing a biofortification level of over 120% compared to the control. Furthermore, nano magnesium at 200 ppm led to a higher yield of more than 300%. Overall, these results show that nano-fertilizers can substantially enhance the nutritional quality of crops, providing a promising alternative to traditional fertilizers for improving crop sustainability and value. Similarly, Wang et al. [138] reported that the application of iron (ferric) phosphate nanomaterials enhanced the nutritional value of tomatoes by improving the accumulation of flavonoids, which are recognized for their health benefits and the ability to prevent chronic diseases. The study discovered that the application of 50 mg Kg^−1^ of soil led to high levels of plant hormone auxin (AII), which in turn resulted in more efficient absorption of essential nutrients such as manganese, magnesium, and iron.

In addition, the study conducted by Al-Saif et al. [139] aimed to explore the impact of using zinc oxide nanoparticles (ZnONPs) and boron oxide nanoparticles (B203NPs) on the phytochemical and physiochemical properties of the Wonderful pomegranate trees. The method utilized includes applying ZnONPs and B203NPs at concentrations of 0.20, 0.50, and 1 g/L during three development stages: full bloom, six weeks after full bloom, and one month before harvest. The results demonstrated significant improvements in the physical properties of the fruits, such as firmness, weight, and color of the fruits, resulting in more appealing produce and marketable produce. Moreover, the treated fruits demonstrated high levels of beneficial bioactive compounds, including anthocyanins, total phenolics, ascorbic acid, flavonoids, and enhanced antioxidant activity in the pomegranate juices. Notably, the optimal results were observed with 0.50 and 1 g/L concentrations of ZnONPs and 1 g/L of B203NPs, highlighting the significance of precise doses in optimizing the quality and nutritional content of the fruit.

López-Vargas et al. [140] demonstrated that the foliar application of copper nanoparticles led to an increase in the firmness of tomato fruits and increased vitamin C, lycopene, and antioxidant capacity in tomato fruits. In addition, the activity of essential enzymes such as superoxide dismutase (SOD) and catalase (CAT) was improved; these enzymes play a significant role in reducing oxidative stress. Similarly, the application of copper nanoparticles to bell peppers under saline stress conditions increased yellow carotenoids, phenols, glutathione, and flavonoids in the fruits [17]. Wang et al. [141] reported that the use of sulfur nanoparticles in tomato plants led to a significant improvement in nutritional quality. The improvement was observed in the increased content of the essential nutrients, including magnesium, iron, calcium, and copper, in the tomato fruits. Thus, researchers have reported that the higher amount of beneficial bioactive compounds present in fruits improves their nutritional value by providing extra health benefits. The presence of bioactive compounds such as vitamins, minerals, polyphenols, carotenoids, and flavonoids in fruits enhances their therapeutic and medicinal properties. These compounds provide anticancer, antioxidant, and anti-inflammatory properties, which improve the nutritional value of fruits [142,143,144].

Another study by Yue et al. [145] found that the application of manganese ferrite nanomaterials resulted in an increase in beneficial compounds, including glucose-6-phosphate, rutin, phenylalanine, and vitamin C, making them healthier to consume. In addition, the manganese ferrite nanomaterials increased the chlorophyll content by 20% in tomato leaves. These nanomaterials upregulate genes responsible for transporting sucrose. The higher levels of beneficial compounds such as vitamin C, glucose-6-phosphate, rutin, and phenylalanine lead to higher nutrient density, providing sufficient concentrations of essential mineral elements and organic molecules for human nutrition [23]. Researchers have reported that higher chlorophyll content in leaves can result in higher nutritional value in crops owing to the presence of bioactive compounds such as chlorophylls [146]. Yang and Wang [147] evaluated the impact of iron oxide nanoparticles and fulvic acid-coated iron oxide nanoparticles on soybeans plants. The findings of the study revealed that the foliar application of iron oxide nanoparticles led to 2–4 times higher iron concentration in the soybean’s shoots compared to the soil application. In addition, the application of fulvic acid-coated iron oxide nanoparticles enhanced the absorption of essential nutrients such as zinc and potassium. The fulvic acid-coated iron oxide nanoparticles stimulated biological nitrogen fixation, resulting in the formation of more root nodules, ultimately improving nutritional value. Increasing essential nutrients can enhance the nutritional value of soybeans [148]. Overall, the utilization of nano-fertilizers increases agricultural productivity and significantly improves the nutritional value and quality of crops, providing a potential solution to meet dietary requirements and enhance public health.

## 3. Conclusions

The efficacy and practical applicability of nano-fertilizers in agriculture depend heavily on their physical properties, including size, charge, shape, surface area-to-volume ratio, crystalline structure, and agglomeration. Understanding the properties of nano-fertilizers is critical to unlocking their full potential, as they significantly influence nutrient use efficiency and crop performance. For instance, plants can easily absorb nano-fertilizers due to their small particles, and their controlled release mechanisms precisely deliver nutrients when needed. Efficient nutrient delivery systems can aid in minimizing waste and reducing the ecological footprint of agricultural practices.

The impact of nano-fertilizers on improving the physiological properties and nutritional value of plants cannot be overlooked. As the population continues to rise and the demand for food increases, nano-fertilizers have the potential to provide nutritious, safe, and sufficient food to feed the population and address the issue of food insecurity. Therefore, by tailoring their physical properties and addressing formulation challenges, their full potential can be harnessed, and more sustainable and efficient agricultural systems can be created. While the potential of nano-fertilizers is substantial, realizing their advantages necessitates continued innovation and interdisciplinary research. The tailored design of nano-fertilizers can overcome existing agricultural challenges, thereby improving food security and promoting environmental sustainability.

## 4. Future Prospects

Extensive field trials and long-term studies should be conducted to assess the real-world efficacy of nano-fertilizers in various agro-climatic regions. This will aid in comprehending their influence on crop performance over multiple growing seasons. Although the benefits of nano-fertilizers are evident, it is important to thoroughly examine their potential impacts on the environment and human health. Research should aim to determine safe concentration thresholds and develop guidelines for their use. In addition, it is vital to assess the economic viability of nano-fertilizers before their widespread adoption. Future studies should perform cost-benefit analyses, accounting for the long and short economic impacts on all types of farmers. Collaboration research between agronomists, soil scientists, and nanomaterial scientists should be implemented to advance nanotechnology in agriculture.

## 5. Recommendations

- Cytotoxicity studies should be conducted prior to the application of nano-fertilizers, as several researchers have expressed concerns about the potential toxicity of nanoparticles due to their small particle size and large surface area, which can lead to increased reactivity. The effects of accidentally ingesting the residue of nanoparticles from plants remain unknown.
- Further studies should focus on investigating the impact of different nanoparticle shapes on nutrient uptake and plant growth. It is important to determine the most suitable nanoparticle shape for different plant species in order to enhance the effectiveness of nano-fertilizers and improve overall plant performance. Additionally, researchers should aim to synthesize nano-fertilizers with specific shapes designed to meet the requirements of different plant species.
- There is scarce information about what happens when nanoparticles enter plant cells or tissues, making it uncertain whether they aggregate into agglomerates. Researchers should develop sensors to monitor nanoparticle behavior once inside plant cells or tissues. This will assist in tailoring the properties of nano-fertilizers to enhance their efficacy.

## Figures and Tables

**Table 1:** The relationship between particle size and uptake by plant.

Crop Type	Nanoparticle Type	Nanoparticle Size	Effect on Nutrient Uptake	Reference
Watermelon	AgNPs	20 nm	63.8% of the NPs were absorbed 38.2% were found on the outer surface of the leaves	[53]
		60 nm	21.7% of the NPs were absorbed 8.3% accumulated on the outside surface	
*Nicotiana xanthi*	AgNPs	3.5 nm	NPs penetrated the cell wall	[54]
		18 nm	NPs did not enter the roots and instead gathered on the outer surface.	
Soybean	CuONPs	25 nm	Exhibited high nutrient uptake	[50]
		50 nm	Demonstrated lower nutrient uptake compared to the CuONPs 25 nm	
Wheat	SeNPs	40 nm	The absorption was 1.8–2.2 times higher than SeNPs 140 nm and 240 nm.	[48]
		140 nm	The absorption was 1.8–2.2 times lower than SeNPs 40 nm	
Cucumber	Ceria NPs	7 nm	Exhibited higher uptake of ceria NPs	[6]
		25 nm	Demonstrated lower uptake of ceria NPs	
*Allium porrum*	Water-suspended fluorescent polystyrene NPs	43 nm	The NPs were able to penetrate through the stomatal pores	[38]
		1100 nm	The NPs were not able to penetrate They accumulated on the outer surface	

**Table 2:** The slow-release mechanism of nano-fertilizers in comparison to their bulk materials.

Type of Fertilizers	Nanoparticle Material Release Time	Bulk Material Release Time	Reference
Nitrogen-based fertilizer	1000 h (about 1 and a half months)	500 h	[64]
Nitrate nitrogen fertilizer	Exceeded 50 days	10–12 days	[71]
APTMS-modified zeolite	120 min (2 h)	10 min	[72]
urea-hydroxyapatite fertilizer	60 days	30 days	[73]
Urea-loaded polycaprolactone nanocomposite	>90 h	<25 h	[74]
Phosphate fertilizer	40–50 days	10–12 days	[75]
DAP	60 days	15 days	[76]

**Table 3:** Different ways in which nano-fertilizers are designed or engineered to release nutrients.

Control-Release Fertilizers	Properties	Reference
1. Slow-release fertilizer	Slow-release fertilizer utilizes nanocapsules for controlled nutrient release. Nanocapsules provide a gradual and sustained supply of nutrients over a predetermined duration.	[80]
2. Quick-release fertilizer	Nanoparticles coated with a protective shell are utilized in quick release fertilizers. The shell is made of a material designed to break down under certain conditions. The trigger for activation can involve physical contact with a surface, such as a plant’s leaf or the soil. Quick-release fertilizer is advantageous when there is an immediate need for nutrient replenishment.	[10]
3. Specific-release fertilizer	The fertilizer is enclosed in nanoscale particles, typically with a protective shell to delay its release. The nanoparticle shell is engineered to exhibit controlled release by selectively responding to specific chemical molecules in the surrounding environment. Upon contact with the targeted chemical molecules, a chemical interaction occurs. This interaction may impact the structural integrity of the nanoparticle shell. The chemical interaction leads to the breakdown of the nanoparticle’s protective shell. Upon shell rupture the contents of the nanoparticle, including fertilizers or active substances, are released into the surrounding environment.	[11]
4. Moisture-release fertilizer	The moisture-release fertilizer is designed to facilitate the controlled degradation of nanoparticles, This results in the gradual release of nutrients upon exposure to water.	[10,11]
5. Heat-release fertilizer	The heat-release fertilizer utilizes nanoparticles to facilitate the controlled release of nutrients. This innovative approach allows for the gradual release of nutrients when the surrounding temperature surpasses a specific threshold.	[10]
6. pH-release fertilizer	The pH-release fertilizer employs nanoparticles that exclusively undergo degradation within a specific acidic or alkaline environment	[11]
7. Ultrasound release	The nanoparticle undergoes rupture upon exposure to an externally applied ultrasound frequency.	[80]
8. Magnetic release	Magnetic release involves the rupture of a magnetic nanoparticle upon exposure to an external magnetic field.	[10]

**Table 4:** The relationship between nanoparticle shape and plant performance in various crop species.

Crop	Nanoparticle Type	Concentration	Nanoparticle Shape	Germination (%)	Plant Development	Reference
Lentil	AuNPs	5 ppm	Spherical	There was no significant difference observed	Plant height = 17.90 cm Number of leaves = 14.33 Biomass production = 6.70 gm	[90]
		10 ppm		No significant difference observed	Plant height = 23.23 cm Number of leaves = 17.67 High biomass production = 8.20 gm.	
		25 ppm		26.7	Plant height = 15.10 cm Number of leaves = 13.33 Biomass production = 5.57 gm	
		50 ppm		53.3	Plant height = 12.90 cm Number of leaves = 10.33 Biomass production = 3.80 (gm)	
		100 ppm		66.7	Plant height = 10.77 cm Number of leaves = 8.00 Biomass production = 2.77 gm	
*Phaseolus vulgaris*	AgNPs	15 mg L^−1^	Spherical	100	Moderate effect observed for all studied parameters	[91]
		30 mg L^−1^		100	Moderate effect observed for all studied parameters	
		60 mg L^−1^		100	Higher shoot growth Higher plant height High number of leaves	
		120 mg L^−1^		93.33	Higher root growth observed High root length	
		240 mg L^−1^		80	Lower shoot and root growth	
		480 mg L^−1^		73.33	Lower shoot and root growth Lower root length Less number of leaves Lower plant height	
*Green pea*	AgNPs	20 mg L^−1^	Spherical	98	High root length of 20 cm High root fresh weight Lower root deformation	[92]
		40 mg L^−1^		96	Lower root fresh weight	
		80 mg L^−1^		87	Moderate effect for studied parameters	
		160 mg L^−1^		85	Lower root length of 10 cm Lower root fresh weight High root deformation	
Blackgram	ZnONPs	100 mg L^−1^	Spherical	67	Lower shoot length Lower root length	[93]
		200 mg L^−1^		68	Moderate shoot and root length	
		300 mg L^−1^		69	Moderate shoot and root length	
		400 mg L^−1^		70	Moderate shoot and root length	
		500 mg L^−1^		72	Moderate shoot and root length	
		600 mg L^−1^		74	Higher shoot length Higher root length	
Wheat	ZnONPs	10 mg L^−1^	Spherical	78	Lower plant fresh biomass Lower leave length	[94]
		25 mg L^−1^		80	Moderate results for all parameters studied	
		50 mg L^−1^		80	Higher fresh biomass Higher number of roots Higher leave length	
		100 mg L^−1^		80	Moderate results for all parameters studied	
*Brassica oleracea* var italic	ZnONPs	50 µg L^−1^	Spherical	87.5	Lower plant height = 16.6 cm	[95]
		100 µg L^−1^		100	-	
		200 µg L^−1^		87.5	Higher root length	
		400 µg L^−1^		87.5	Plant height = 19.8 cm	
		800 µg L^−1^		87.5	Plant height = 20 cm Higher number of leaves = 8.66 Higher leaf area = 62.48 cm^2^ Higher root length = 57.44 cm	
		1000 µg L^−1^		87.5	Higher plant height = 20.33 cm	
green gram *Vigna radiata*	ZnONPs	100 mg L^−1^	Rod	Lower germination% compared to the other concentration Lower germination% compared to the other concentration	-	[96]
		200 mg L^−1^		Higher germination% compared to the other concentration	Higher shoot length = 16 cm Higher root length = 6 cm	
		300 mg L^−1^		Lower germination% compared to the other concentration	-	
		400 mg L^−1^		Lower germination% compared to the other concentration	-	
Groundnut	ZnONPs	500 mg Kg^−1^	Rod	58	Lower shoot length = 18.40 cm Lower root length = 15.67 cm	[97]
		750 mg Kg^−1^		63	Shoot length = 19.88 cm Root length 17.98 cm	
		1000 Kg^−1^		75	Higher shoot length = 20.98 cm	
		1250 mg Kg^−1^		71	shoot length = 20.28 cm Root length = 17.98 cm	

## Data Availability

No new data were created or analyzed in this study.
